# Differential sources of host species heterogeneity influence the transmission and control of multihost parasites

**DOI:** 10.1111/ele.12122

**Published:** 2013-05-28

**Authors:** Daniel G Streicker, Andy Fenton, Amy B Pedersen

**Affiliations:** 1 Odum School of Ecology, University of Georgia140 E. Green St., Athens GA 30602; 2 Institute of Integrative Biology, University of LiverpoolCrown Street, Liverpool, L69 7ZB, UK; 3 Centre for Infection, Immunity and Evolution, School of Biological Science, University of EdinburghKings Buildings Ashworth Labs, Edinburgh, EH9 3JT, UK

**Keywords:** Coccidia, community epidemiology, helminth, management, parasitism, species heterogeneity, super-shedder, susceptibility

## Abstract

Controlling parasites that infect multiple host species often requires targeting single species that dominate transmission. Yet, it is rarely recognised that such ‘key hosts’ can arise through disparate mechanisms, potentially requiring different approaches for control. We identify three distinct, but not mutually exclusive, processes that underlie host species heterogeneity: infection prevalence, population abundance and infectiousness. We construct a theoretical framework to isolate the role of each process from ecological data and to explore the outcome of different control approaches. Applying this framework to data on 11 gastrointestinal parasites in small mammal communities across the eastern United States reveals variation not only in the magnitude of transmission asymmetries among host species but also in the processes driving heterogeneity. These differences influence the efficiency by which different control strategies reduce transmission. Identifying and tailoring interventions to a specific type of key host may therefore enable more effective management of multihost parasites.

## Introduction

The majority of parasites, including many that cause emerging human, domestic animal and wildlife diseases, infect multiple host species (Cleaveland *et al.* 2001; Pedersen *et al.* 2005; Woolhouse & Gaunt 2007). However, differences in host species’ abundance, exposure and susceptibility imply that each species is unlikely to contribute equally to parasite transmission (Haydon *et al.* 2002; Altizer *et al.* 2003). If heterogeneities among host species are severe, certain species may contribute disproportionately to transmission and become a ‘key host’, responsible for long-term parasite persistence and infection of sympatric host species. Many studies have revealed the presence of key host species among the communities of potential hosts. For example, because of heterogeneities among the mammalian hosts of *Borrelia burgdorferi*, the causative agent of Lyme disease, shifts in host community composition that reduce the number of tick bites on highly infectious white-footed mice (*Peromyscus leucopus*) can reduce Lyme disease prevalence in ticks and humans (LoGuidice *et al.* 2003). Similarly, rabies virus infections in wild carnivores in the Serengeti depend on viral maintenance by domestic dogs, so vaccinating dogs is expected to alleviate epizootics in wildlife (Lembo *et al.* 2007).

Such studies generally point to a small subset of host species as dominating the transmission dynamics of multihost parasites. Consequently, interventions such as vaccination, culling or sterilisation commonly target single species rather than all infected hosts (Jolles *et al.* 2005; Donnelly *et al.* 2006; Kaare *et al.* 2009). Being able to identify these key hosts is essential in determining which host species to target (Haydon *et al.* 2002; Caley & Hone 2004; Fenton & Pedersen 2005). However, it is less well recognised that key hosts can arise through separate processes. For instance, the importance of dogs for rabies transmission is driven at least partly by their higher population densities relative to other carnivore species (Lembo *et al.* 2008), but West Nile virus transmission around Washington D.C. is dominated by the presence of a relatively rare, but highly infectious bird, the American robin (Kilpatrick *et al.* 2006).

The underlying drivers of interspecific heterogeneities may influence the community dynamics and control of multihost parasites. If key hosts arise through infectiousness rather than population density, optimal control strategies might focus on identifying and managing highly infectious host species rather than the most commonly infected host species. In contrast, if key hosts arise through high prevalence or abundance, the success of control might be proportionate to the number of animals treated. Finally, the added diagnostic costs of test-and-treat or test-and-cull programs, e.g. bovine tuberculosis in African buffalo; Jolles *et al.* (2005), that target only infected individuals might yield only trivial gains over untargeted control for certain drivers of host heterogeneity, but may be highly fruitful for others.

Understanding the impact of host species heterogeneity on the transmission dynamics and control of multihost parasites is limited by challenges in quantifying the extent and sources of the various processes that contribute to the identity of key hosts. In addition, few data sets are available to distinguish sources of heterogeneity and quantify their impact on transmission. Here, we first identify three ways in which a host species may become a key host and describe a mathematical framework to partition the contribution of each process. Second, by comparing the efficacy of treating infected vs. random individuals under each key host type, we show that very different implications for control arise from these different processes. Finally, we apply these concepts to empirical data sets on gastrointestinal parasite infections in wild small mammal communities across the eastern United States to estimate the contribution of each host species to parasite transmission, identify the processes underlying the key hosts’ dominance of transmission and predict the impact of hypothetical control strategies on parasite transmission.

Overall we show, both theoretically and empirically, that host species make very different contributions to parasite transmission, and may do so through a variety of processes. Being able to quantify these processes, identify key hosts and determine what kinds of key hosts they are may enable a better understanding of how parasites are maintained in multihost communities and aid the development of more successful disease management strategies.

## Conceptual Framework for Understanding Host Species Contributions to Parasite Transmission

A parasite's fitness can be measured by its basic reproduction number, R_0_, the number of new infections (for a microparasite, such as a virus or bacteria) or new adult parasites (for a macroparasite, such as a helminth or ectoparasite) arising from a primary infection in a wholly susceptible host population or community (Anderson & May 1991). In a multihost species context, each host species *i* contributes a proportion (r_0*,i*_) to the total R_0_. Host species *i* would be formally classified as a key host if r_0*,i*_ exceeds a threshold value, *T*.

In practical terms measuring R_0_, or individual host contributions to R_0_, from field data can be difficult because estimates of both within- and between-species transmission rates are rarely available (Dobson 2004). The relative magnitude of these transmission rates will depend, among other things, on the type of parasite involved, its route of transmission and host factors such as behaviour, territoriality and spatial overlap that can reduce mixing among species. Although it may be possible to approximate relative degrees of cross- and within-species transmission from measures such as home range overlap, it is still a major challenge to translate these into contributions to R_0_. However, for parasites such as helminths, vector-borne parasites and some directly transmitted bacteria and viruses, it is often possible to measure each host species’ contribution to the parasite's overall transmission pool as the number of infective stages or infected vectors produced by each host species (Ferrari *et al.* 2004; Kilpatrick *et al.* 2006; Matthews *et al.* 2009). Here, we use these measures of each species’ contribution to the total infectious pool as proxies for their contributions to the parasite's total R_0_, using a hypothetical macroparasite system (e.g. a parasitic helminth that transmits via a free-living infective stage) to illustrate our concepts.

In what follows we define the following symbols: *H*_*i*_ is the total number of individuals (infected or not) of host species *i*, *I*_*i*_ is the number of individuals of host species *i* infected by the parasite, *p*_*i*_ is the prevalence (proportion infected, *I_i_/H_i_*) of host species *i* and *λ*_*i*_ is the *per capita* number of parasite eggs shed by an infected individual of host species *i* throughout the duration of infection. The overall contribution to the parasite's infectious pool by host species *i* is *n*_*i*_ = *H*_*i*_*p*_*i*_*λ*_*i*_. If there are *N* host species in the community then the total input to the parasite's infectious pool is as follows: 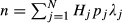, and the relative contribution of host species *i*, *π*_*i*_, is as follows:(1)
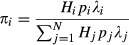


Host species *i* is classified as a key host if *π*_*i*_ > *T*, which is a more practical definition of host contributions than the requirement r_0,*i*_ > *T*. From eqn 1, there are three ways in which host species *i* can make a substantial contribution to the total infectious pool: (1) by being highly abundant (large *H*_*i*_; a ‘super-abundant’ key host), (2) by having a high prevalence of infection (large *p*_*i*_; a ‘super-infected’ key host) and/or (3) by producing a large number of infective stages per infected individual (large *λ*_*i*_; a ‘super-shedder’ key host). Note we use the term ‘super-shedder’ as a species-level parameter (i.e. a species that, on average, sheds a large number of infective stages) rather than the intraspecific use describing variation among infected individuals within a species). In Box 1, we describe each mechanism in turn and illustrate how the degree of asymmetry in each process in the key host relative to the average in the host community (defined as 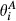, 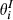 and 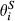, respectively, for super-abundant, super-infected and super-shedding key hosts) may be quantified from ecological data.

**Box 1 Description and quantification of the three asymmetries underlying host heterogeneity**

Equation 1 shows that a host species can make disproportionate contributions to the total infectious pool by being ‘super-abundant,’ ‘super-infected’ and/or a ‘super-shedder.’ Here, we derive each form of asymmetry and show how these can be combined to calculate the total contribution (*π*_*i*_) of each host species *i* to transmission dynamics at the community level.

**Super-abundant hosts**

The degree of asymmetry in host abundance for host species *i* is:
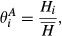

where 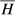 is the average number of individuals per host species across the whole community 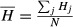 (including species *i*). Host species *i* shows abundance asymmetry if 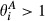 (i.e. if it is more abundant than expected based on the community average).

**Super-infected hosts**

For host species *i*, the degree of infection asymmetry is:
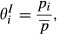

where 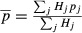, which is the total number of infected hosts in the community divided by the total number of individuals (i.e. the average prevalence of infection across the whole host community, regardless of species identity). Host species *i* shows infection asymmetry if 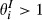 (i.e. if it is infected more often than expected based on the community average prevalence).

**Super-shedder hosts**

For host species *i*, the degree of shedding asymmetry is:
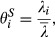

where 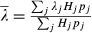, which is the total number of infective stages shed by all infected host individuals in the community divided by the total number of infected hosts (i.e. the average *per capita* rate of shedding across the whole host community, regardless of species identity). Host species *i* therefore shows shedding asymmetry if 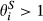 (i.e. if its production of infective stages per infected host is greater than expected based on the community average). Note that this is a species-level use of the term ‘super-shedder,’ describing disproportionate shedding of infective stages by one species relative to the average of the total host community.

**Total contribution of host species *i* to the parasite's infective pool**

Given the above, we can rewrite eqn 1 as:
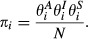

That is, the relative contribution of host species *i* to the parasite's total transmission pool is proportional to the product of that species’ abundance, infection and shedding asymmetries. For host species *i* to be a key host (*π*_*i*_ > *T*  ) at least one of these asymmetries must considerably exceed 1. In other words, it either needs to be much more abundant than other hosts in the community, and/or be infected more than expected based on the community average (e.g. be more exposed to the parasite, or more susceptible to it), and/or shed more infective stages into the environment (e.g. be a highly physiologically suitable host in terms of parasite reproduction).

**Accounting for differential capture probability of host species**

The estimated relative contributions of each host species to overall parasite transmission may be biased by differences in the probability of capturing and sampling each host species in the community. For example, species with low capture probabilities might be underrepresented, so their role in parasite transmission may be underestimated. When capture probabilities can be assessed through mark-recapture sampling, e.g. Williams *et al.* (2002), it is straightforward to take these differences into account. Specifically, if the *per capita* probability for species *i* of being trapped is *a*_*i*_ then the number of individuals observed of species *i* (*h*_*i*_) is *H*_*i*_*a*_*i*_, where *H*_*i*_ is the true abundance of that host species. Hence, *H*_*i*_ in the above theory can be replaced throughout by *h*_*i*_/*a*_*i*_.

To explore the robustness of our empirical findings to unknown variation in sampling probabilities, we assigned random capture probabilities to each host species and calculated the efficacy of control under untargeted, targeted and random removal plans. Capture probabilities were selected from a uniform distribution from 0.1 to 0.6 to encompass typical values estimated for small mammals (Hammond & Anthony 2006). Iterations (*n *=* *100) of this variation in capture probability show that decisions on which control strategy would be favoured are often possible even with the uncertainty arising from variation in capture probabilities (Fig. S6).

### Implications of the different types of key host for control success

To assess the effect of the different key host types on the success of control, we initially assume that host species *i* is a key host through a single, mutually exclusive process. For example, if host *i* is super-abundant (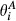 >> 1) then it is not super-infected (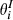 = 1) or a super-shedder (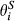 = 1); see Box 1 for details. Later, using theory and our small mammal–parasite data, we relax this assumption and consider key hosts that arise from multiple correlated processes (see Appendix S1, Table S1 and Fig. S1).

For each key host scenario, we explored the efficacy of control that removes a certain number of individuals of species *i* (*C*_*i*_ ). Biologically, removal represents any action that eliminates an individual's contribution to the parasite's infectious pool, such as culling or treatment by deworming, antibiotics or antivirals. We explored two control possibilities for each key host scenario: (1) Untargeted control, where *C*_*i*_ individuals are removed regardless of infection status and (2) Targeted control, where only infected individuals are removed. We quantify the impact of control as the proportion of the parasite's initial infectious pool remaining after control (ξ). This measure differs between targeted (ξ^*T*^) and untargeted (ξ^*U*^) control. For targeted it is:(2)
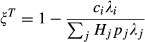
so each *C*_*i*_ individual removed is known to be infected and reduces input to the infectious pool by *λ*_*i*_. For untargeted control, each removed host may or may not be infected, so the effect of removing that individual is weighted by the probability of infection (*p*_*i*_):(3)
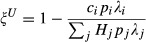


Note that for untargeted control, the maximum number we can remove is *H*_*i*_ (every individual of species *i*) and for targeted control, the maximum number is *H*_*i*_*p*_*i*_ (every infected individual of species *i*).

**Figure 1:**
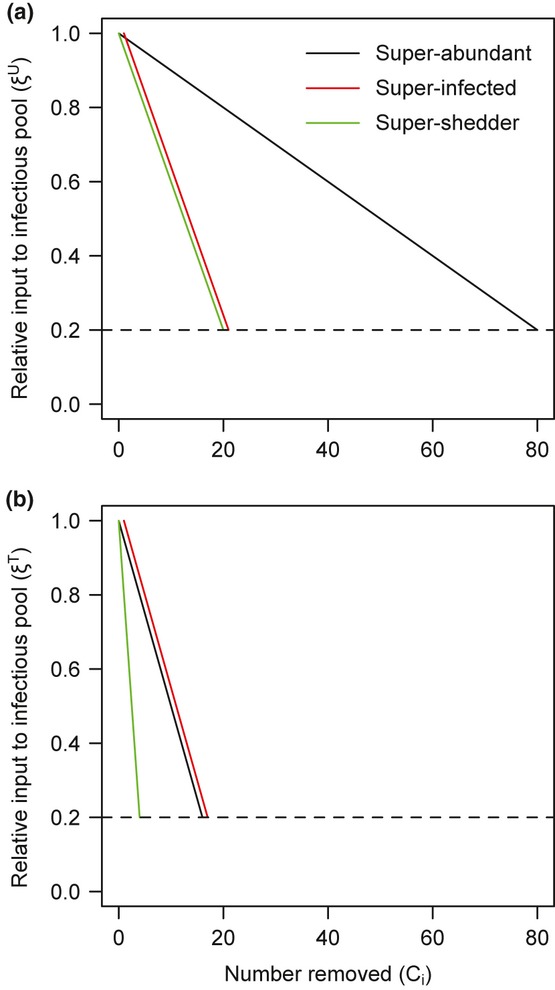
Efficacy of control by (a) untargeted, with respect to infection status, and (b) targeted (of infecteds) removal of *C*_*i*_ individuals of key host species *i*. The efficacy of control is quantified by the proportional reduction in overall contribution to the parasite's transmission pool, ξ^*U*^ and ξ^*T*^ for untargeted and targeted control respectively (eqns e and 3). In each scenario, the host species is assumed to be responsible for 80% of the total contribution to the parasite's infectious pool, and is either a pure super-abundant host (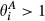,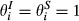; black line), a pure super-infected host (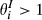,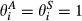; red line) or a pure super-shedding host (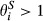,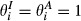; green line). The dashed line represents the maximum reduction in transmission possible by treating only the key host (i.e. the proportion of transmission that is due to the other non-host species). For visualisation, the red lines (super-infecteds) are offset to avoid overlap with super-shedder (panel a) and super-abundant (panel b) key hosts.

The success of each control approach depends on the mechanism by which the key host dominates transmission. Under untargeted control, far greater effort is required to control parasites that have super-abundant key hosts compared to super-infected or super-shedders (i.e. for a given effort, 
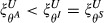
; Fig.1a). The low efficacy of untargeted control for super-abundant key hosts reflects the low probability of randomly removing an infected host in a large population with low prevalence. For super-infected and super-shedding key hosts, either most removals decrease the input to the infectious pool due to high prevalence (super-infected hosts) or infected hosts with disproportionate parasite shedding are removed in proportion to their frequency in the population (super-shedding hosts); in both cases untargeted removal is efficient. In contrast, when it is possible to target infected hosts, super-abundant and super-infected key hosts are equally difficult to control, but removing super-shedder key hosts drastically improves control prospects (i.e. 
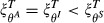
; Fig.1b). The equivalence of super-abundant and super-infected key hosts under targeted control occurs because there are either many individuals but relatively few infected (super-abundant case) or relatively few individuals but many of them infected (super-infected case).

As expected, targeted removal of infected individuals always reduces input to the infectious pool more than untargeted removal; however, the benefits varied widely across the types of key host species. Specifically, compared with untargeted control, targeting infected hosts substantially reduces the number of removed hosts needed to deplete the infectious pool for super-abundant and super-shedding key hosts, but provides minimal benefits when key hosts are super-infected (Fig.1a,b). By far the best scenario for control is targeted control of a super-shedding key host (Fig.1b, green line). Here, relatively few individuals are infected (compared with the other scenarios) and each contributes a great deal to the infectious pool, meaning rapid reductions are achieved by the removal of relatively few individuals.

## Empirical Application: Gastrointestinal Parasites of Small Mammal Communities

The above theory shows that host species’ contributions to parasite transmission can vary depending on the relative abundances, exposures and susceptibilities to infection and suitability for parasite replication. Different combinations of these processes mean that host species can be key hosts due to a variety of different mechanisms that might require different control strategies. But, how do these concepts apply to natural communities where the strength of interspecific heterogeneities may vary and the processes that underlie key hosts may not be independent?

We applied the metrics described above to data sets of 11 gastrointestinal parasites studied in spatially and temporally overlapping small mammal communities in the eastern United States. Gastrointestinal parasites are a good test of this framework because they are common, taxonomically diverse, vary across a continuum of host specificity and utilise a variety of transmission strategies. Moreover, the quality of individual hosts for parasite fitness can be quantified using faecal egg or oocyst counts as an index of shedding of infective stages that scales positively with the number of new infections (Keymer & Anderson 1979; Ferrari *et al.* 2004). The ability to estimate prevalence, relative abundance and relative infectiousness allows us to identify key hosts, tease apart the mechanisms by which they dominate transmission and predict the consequences for control.

## Materials and Methods

### Study sites, host and parasite data

**Figure 2:**
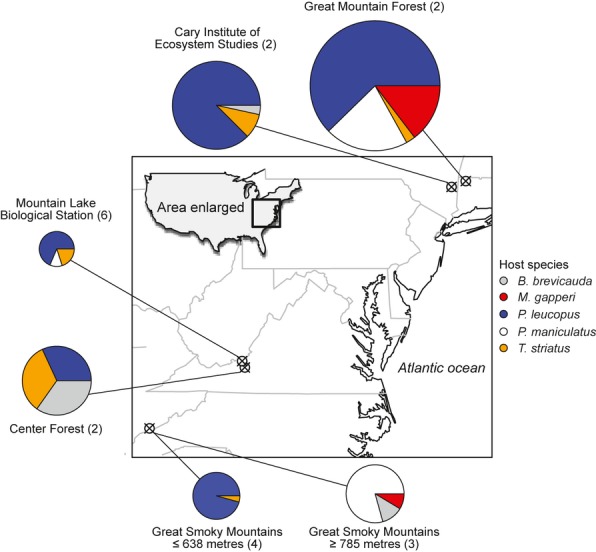
Map of field sites in the eastern United States. The number of grids trapped is indicated in parentheses. Pie charts display the host species composition of each site, with pie diameters proportional to the total number of animals captured (range: 35–130).

We collected data on small mammal (*Rodentia* and *Soricomorpha*) community composition and gastrointestinal parasite prevalence and egg/oocyst burdens across 19 grids in six sites in Virginia, Tennessee, New York and Connecticut (Fig.2). In each site, animals were captured for 2–3 consecutive nights and faecal samples were collected to diagnose gastrointestinal parasite infection and to quantify parasite eggs/oocysts per gram of faeces (details provided in Appendix S2). We were unable to identify all parasite eggs to species and thus only included 11 relatively common parasite species or pseudo-species (five nematodes, three cestodes and three coccidia) for which we were confident in our consistent identification and were found in at least 10% of individuals of one host species (Table S2).

We use this data set to illustrate the utility of our framework for elucidating the mechanisms underlying heterogeneities in disease transmission and informing decisions on control strategies. We acknowledge that our data set lacks some information that would be needed to conduct a full analysis of these communities to control parasites of health or economic concern. First, because the data were collected over a short period of time at each location, long-term patterns of parasite shedding or durations of infections are unknown. For simplicity, we therefore assume that the observed rates of parasite shedding reflect the true relative contributions to each parasite's infective pool. Second, capture probabilities of the different host species may vary (Hammond & Anthony 2006), which might influence assessments of key hosts. As we could not estimate capture probabilities, we provide a theoretical extension to our framework to include such data and conduct simulations to assess the sensitivity of our findings to unknown variation in capture probabilities among host species (Box 1).

### Statistical analysis of host species effects on parasite transmission

We used generalised linear mixed models (GLMMs) to test whether host species differed in prevalence and parasite shedding. We also included two measures of host density as fixed effects: (1) the density of each host species (‘species density’), to identify the effect of abundance of the same species and (2) the total small mammal host density, to identify responses in parasite transmission driven by the overall host community density (‘small mammal density’). Density was estimated as the minimum number of animals alive, scaled by trapping effort and was log_10_ transformed. Prevalence models assumed a binomial response, and models of parasite shedding used a negative binomial response. The site and month of capture were treated as random effects to control for temporal and spatial non-independence. Because the quantity of faeces shed by each host species may differ greatly among host species, we repeated these analyses after multiplying egg burdens by the basal metabolic rate or body mass of host species, using data compiled by White *et al.* (2009). Models were fit in the *glmmADMB* package of R and simplified by sequential term removal and likelihood ratio tests (Zuur *et al.* 2009; R Development Core Team 2011; Skaug *et al.* 2012).

To identify whether host species were super-abundant, super-infected or super-shedding, values of *θ*^*A*^, *θ*^*I*^ and *θ*^*S*^ for each host species of each parasite were calculated as described in Box 1. We defined key host species as those that contributed more to the overall transmission pool than the remaining host community combined (*π*_i_ > 0.5). To quantify the degree of variability across the host community in contributions to parasite transmission, we adapted Pielou's evenness index, *J’* (D/D_max_), where D is the Shannon diversity index calculated from the relative contribution of each host species to parasite transmission (*π*_i_, see Box 1) as – ∑ *π*_i_ *ln π*_i_. D_max_ is the theoretical maximum if all host species contribute equally to transmission (D_max_ = ln *N*) (Pielou 1966). Values of *J’* are constrained between 0 (complete dominance by a single species) and 1 (equal contributions of all infected host species).

## Results

### Host species effects on parasite infection and shedding

Across 19 grids, we captured 468 small mammals of eight species. The most common species were *P. leucopus* (*n *=* *254), *P. maniculatus* (*n *=* *83), *Tamias striatus* (*n *=* *49), *Blarina brevicauda* (*n *=* *35) and *Myodes gapperi* (*n *=* *24), accounting for 95.1% of all mammals captured. Most individuals (83.9%, 270/322) were infected by at least one of the 11 common parasites. All further analyses were restricted to focal host and parasite species.

All parasites infected multiple species (Table S2), but host species contributed differentially to the total number of infected individuals for all parasite species except *Pterygodermatites* A and Cestode A (χ^2^ test: *P *<* *0.05 for all others), with single-host species frequently accounting for more than 70% of infected individuals. The prevalence of infection varied significantly among host species for all parasites except *Eimeria arizonensis* A, *E. arizonensis* B and *Hymenolypis dimunata*, even after controlling for spatiotemporal heterogeneity and variation in relative host densities among sites (Table1). Similarly, GLMMs demonstrated significant differences among host species in egg/oocyst shedding for five parasite species, with additional significant host effects in models that included body mass to account for variation in faecal volumes across species (Table1).

**Table 1:** Results of generalised linear mixed models of parasite prevalence and shedding

	Host species		Species density		Small mammal density	
d.f.	P*	d.f.	*P*	d.f.	*P*
Prevalence						
* E. arizonensis* A	1	0.62	1	0.85	1	0.24
* E. arizonensis* B	1	0.33	1	0.62	1	0.54
* E. delicata*	1	**0.04**	1	0.08	1	**0.01**
* A. americana*	4	**<0.001**	1	**0.02**	1	**0.04**
* C. americana*	4	**<0.001**	1	0.37	1	0.19
* Pterogodermatites* A	4	**0.02**	1	**0.05**	1	0.23
* Pterogodermatites* B	4	**<0.001**	1	0.14	1	0.08
* Strongyle* A	4	**<0.001**	1	0.15	1	**0.01**
* Cestode* A	4	**<0.01**	1	**0.03**	1	**0.02**
* H. dimunata*	4	0.87	1	0.28	1	0.39
* H. citelli*	4	**<0.01**	1	0.25	1	0.50
Parasite shedding						
* E. arizonensis* A	1	0.30	1	**<0.01**	1	**0.01**
* E. arizonensis* B	1	0.72	1	0.08	1	0.30
* E. delicata*	1	**<0.001**	1	**<0.001**	1	0.14
* A. americana*	2	0.18	1	0.82	1	0.63
* C. americana*	4	0.52†	1	0.67	1	0.08
* Pterogodermatites* A	4	**<0.001**	1	0.71	1	0.65
* Pterogodermatites* B	4	**<0.01**	1	0.36	1	**0.02**
* Strongyle* A	4	0.28	1	0.43	1	0.42
* Cestode* A	3	**0.01**	1	1.00	1	1.00
* H. dimunata*	4	0.06‡	1	1.00	1	0.30
* H. citelli*	2	**0.05**	1	0.50	1	0.06

* *P* values were calculated from likelihood ratio tests following term removal from full models; all models contained random effects of sampling site and month of sampling; statistical support for terms agreed qualitatively with models including basal metabolic rate and body mass except where otherwise noted. Bold values indicate statistical significance *P* < 0.05.

† Statistically significant in the model including body mass (Likelihood ratio, LR = 17.93, d.f. = 4; *P *=* *0.001).

‡ Statistically significant in the model including body mass (LR = 9.75, d.f. = 4; *P *=* *0.04).

### Super-abundants, super-infecteds or super-shedders?

**Figure 3:**
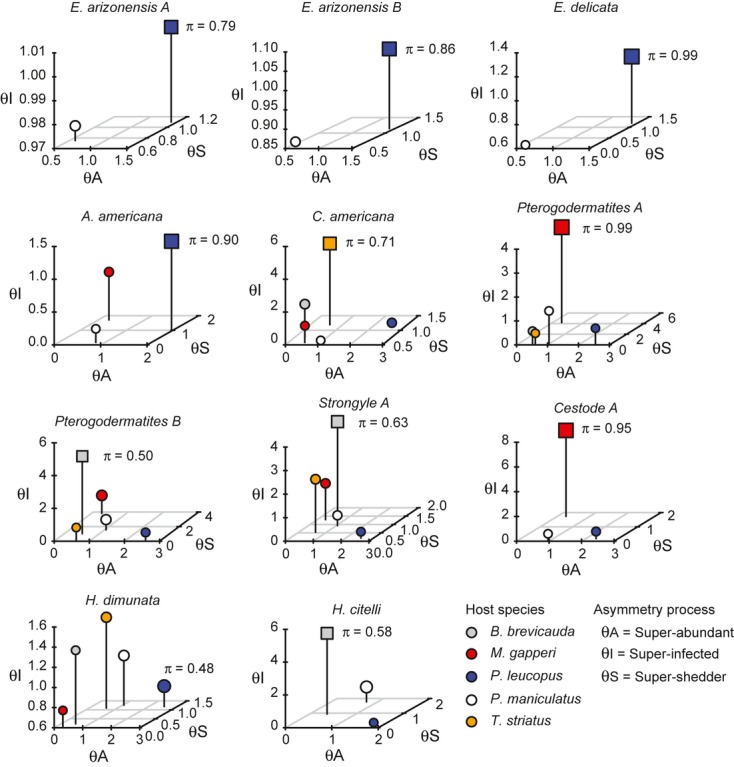
Contributions of three sources of host heterogeneity for 11 multihost parasites. Symbol sizes are proportional to the total contribution of infective stages produced by each host species. Squares indicate the key host species (*π*_i_ > 0.5) for each parasite. Pairwise plots of each source of host heterogeneity are shown in Figs S3–S5.

**Figure 4:**
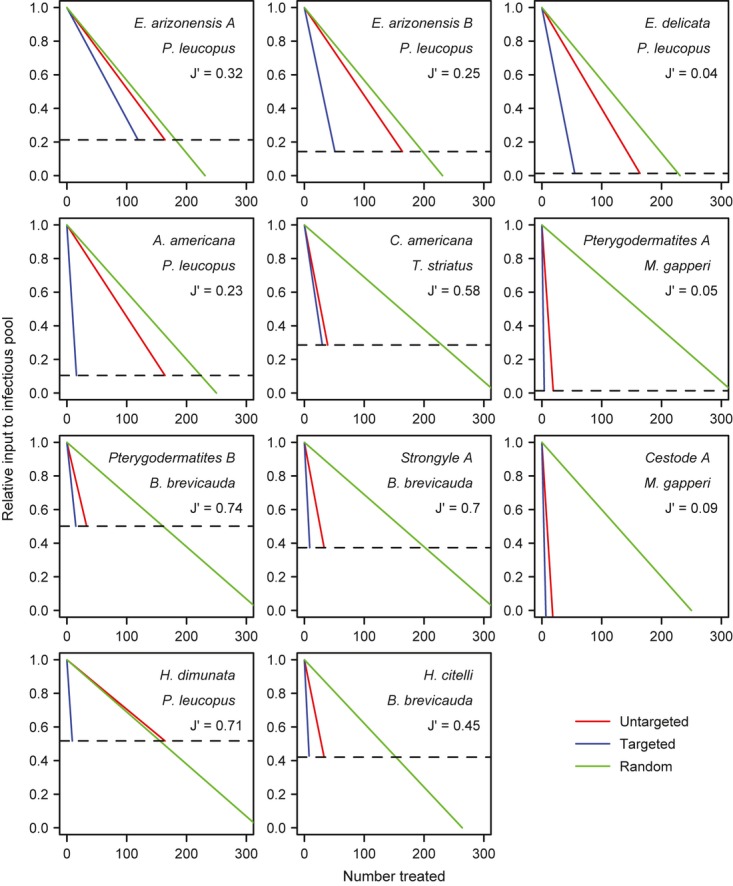
Efficacy of three control strategies for empirical multihost parasites. Each panel shows the expected reduction in the infectious pool size by random removal of individuals regardless of host species (green) and by targeted (blue) and untargeted (red) removal of the most influential host species (shown in the title of each panel). The dashed line shows the maximum reduction that can be achieved by removing all individuals of the key host species (i.e. the proportion of transmission due to non-key host species). The slopes of targeted, untargeted and random lines are given by the following equations: 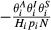, 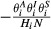 and 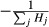 respectively. The *J’* values are Pielou's evenness index and quantify the degree of variability across the host community in contributions to parasite transmission; values of *J’* lie between 0 (complete dominance by a single species) and 1 (equal contributions of all infected host species).

For 10/11 parasites, single-host species produced > 50% of infective stages, meeting our threshold for being ‘key hosts.’ However, among key hosts, contributions to the infectious pool ranged widely from 51 to 99%. At the host community level, variation in the magnitude of heterogeneity in host species’ contributions to parasite transmission was captured by the evenness index, which indicated a range from heavy reliance on a single-host species (e.g. *Pterygodermatites* A, *J’* = 0.05) to relatively equal transmission by several host species (e.g. *H. dimunata*, *J’* = 0.71; Figs.3,4).

Plotting the empirical values of *θ*^*A*^, *θ*^*I*^ and *θ*^*S*^ for each host species revealed the relative contribution of host species’ abundance, prevalence and infectiousness to interspecific heterogeneity in parasite transmission potential (Fig.3, Figs S3–S5). The key host species of some parasites were super-abundant, but with unexceptional prevalence or shedding (e.g. *E. arizonensis* A, *E. arizonensis* B, *E. delicata*, *Aspicularis americana*). The key hosts of other parasites were predominately super-infecteds (e.g. *Capillaria americana*, *Pterogodermatites* B, *H. citelli*), while still others showed a combination of asymmetries, most often being both super-infecteds and super-shedders (e.g. *Pteryogodermatites* A, Strongyle A, Cestode A). Finally, multiple host species contributed relatively evenly to the production of eggs of the tapeworm *H. dimunata*, but through different processes (Fig.3). The three forms of asymmetry were not entirely independent. Super-infecteds also tended to be super-shedders, although this trend was not statistically significant (*r *=* *0.34, *P *=* *0.12; Fig. S2a). Super-abundant hosts were unlikely to also have high infection or shedding asymmetry; however, these were not statistically significant and may reflect similar values of *θ*^*A*^ in each host species across parasite species (Fig. S2b,c).

### Controlling multihost parasites under different processes of host heterogeneity

Applying the theory described above to the empirical data allowed us to explore the efficacy of three hypothetical control strategies for each parasite species: (1) random removal regardless of host species or infection status (‘random’), (2) removal of the host species that contributed the largest proportion of infective stages without respect to infection status (‘untargeted’) or (3) targeting only infected individuals of the species that contributed the largest proportion of infective stages (‘targeted’). In nearly all cases, targeted and untargeted removal reduced the infectious pool more efficiently than random removal (Fig.4). A single exception was *H. dimunata*, where random removal slightly outperformed untargeted control of *P. leucopus*. Here, *P. leucopus* contributed less than 50% of the total infectious pool, and was rarely infected, but super-abundant, so greater reductions could be achieved by treating individuals of other species, which tended to be less abundant, but more commonly infected (Fig.3, Fig.4). For less specialist parasites (i.e. high *J’*), even removal of all individuals of the key host species reduced the infectious pool by only 50–60%. There, multispecies control would be required to substantially suppress the parasite, but this would require removal of many individuals (Fig.4).

Targeted control was generally more effective than untargeted in terms of reducing the parasite pool to a given level by removing fewer individuals; however, importantly, the gains in efficiency ranged from trivial (e.g. *C. americana*) to extensive (e.g. *A. americana;* Fig.4). Comparing eqns 2 and 3 shows that the slopes of these relationships (i.e. the reduction in overall transmission per individual treated) only differ by the prevalence of infection (*p*_*i*_) in untargeted control (eqn 3). Hence, when the parasite is highly prevalent in the key host (*p*_*i*_ →1) targeted and untargeted control is roughly equivalent because untargeted treatments have a very high likelihood of removing infected hosts. However, if prevalence in the key host species is low, but it was either a super-shedder or super-abundant host, there are considerable gains from targeting infected individuals. The success of both host specific control (i.e. targeted and untargeted) strategies scales positively with the degree of shedding asymmetry (*θ*^*S*^); however, the effect is greater with targeted control because each removal of an infected individual has a large effect. Notably, the relative benefits of targeted and untargeted control are insensitive to the degree of abundance asymmetry (*θ*^*A*^). However, efficiency is improved if the total number of host individuals in the community (∑*H*_*j*_) is small, and for targeted control, if the prevalence across the host community is low. Many of these findings are robust to simulated variability in capture probabilities, suggesting that the magnitude of asymmetries are often sufficiently large to be detectable even in the face of substantial uncertainty in species-specific capture success (Fig. S6).

## Discussion

Due to the clear importance of understanding heterogeneities in disease transmission across host communities, many studies focus on identifying reservoir species (Woolhouse *et al.* 1997; Haydon *et al.* 2002; Lloyd-Smith *et al.* 2005). However, it is also important to consider the distinct processes by which host species contribute differentially to parasite transmission and maintenance, a feature that has received relatively little attention. We described a general framework for isolating the components of transmission heterogeneities among the host species of multihost parasites and explored the practical implications for managing infectious diseases. Importantly, variable sources and magnitudes of host heterogeneity yield different expectations for control, such that some parasites require far greater effort and/or qualitatively different strategies to achieve significant parasite reduction.

Consistent with comparative studies that have found most parasites infect multiple host species (Cleaveland *et al.* 2001; Pedersen *et al.* 2005), we found no parasites limited to single-host species (Table S2); however, host species often differed significantly in rates of parasite infection and shedding (Table1). Such differences may reflect variation in parasite exposure because of microhabitat use or physiological differences in susceptibility, the duration of the infectious period or parasite-induced mortality rates. Within the host, high rates of parasite shedding could reflect both increased fecundity of adult parasites or a greater number of moderately fecund adults (Keymer & Hiorns 1986; Kotze & Kopp 2008); however, the consequences are equivalent for the purposes of transmission. Despite infecting multiple host species, the magnitude of host heterogeneity varied dramatically across parasites, indicating a continuum of host specialisation. At the extreme, multihost parasites may depend on a single species for long-term persistence (the ‘maintenance’ host, in the terminology of Haydon *et al.* 2002), although cross-species transmission may still play an important role in shaping the parasite communities of individual hosts. Similar examples of ‘cryptic’ host specificity, where apparently generalist parasites rely heavily on single-host species, have recently been observed for a variety of presumed generalist pathogens including rabies virus in bats (Streicker *et al.* 2010), anther smut fungus in plants of the genus *Silene* (Le Gac *et al.* 2007) and parasitoid flies of insects (Smith *et al.* 2006).

Applying our theoretical framework to data on 11 multihost parasites revealed that key hosts for parasite transmission often arose through multiple processes (Fig.3). Here, super-abundant key hosts were limited to *P. leucopus*, being the most abundant host species in our data set. Thus, for the six parasite species with relatively rare (non-*P. leucopus*) key hosts, the prevalence of infection and shedding rates must be more important (Fig.3). The importance of identifying the mechanisms underlying key hosts is highlighted by our findings that both in theory and practice, efficient management of multihost parasites requires different strategies for each key host scenario (Figs1 and 4) and for scenarios where key hosts arise through a mixture of processes (Fig. S1). We demonstrated three central results. First, while targeting the key host species usually improved the prospects for control, the benefits of doing so were sometimes trivial when control did not target infected individuals. Such non-significant benefits of targeted control were particularly true for parasites with high prevalence in a super-abundant key host. Second, as removal of all individuals of the key host species sometimes yielded only 50–60% reductions in the pool of infective stages, targeting single-host species may be insufficient to eliminate generalist multihost parasites (Figs3 and 4). It is important to note that this result assumes parasite persistence in the absence of the key host. If alternative hosts were mainly infected by transmission from the key host, parasite elimination may be easier than implied here. Field experiments or molecular approaches that quantify the sources of individual infections and the impacts of single-species control might allow for dynamical models of parasite control (Caley & Hone 2004; Streicker *et al.* 2010; Westram *et al.* 2011). Third, we showed that while efforts to identify and remove only infected key hosts can have massive benefits to control some parasites, when key hosts are rare but commonly infected, treating all individuals regardless of infection status is a more practical approach (Fig.4). In these cases, control plans should direct resources to increase treatment rates and reduce investments in diagnostics.

Several layers of additional complexity might be incorporated to broaden the remit of our approach. First, we did not consider behavioural or genetic heterogeneities within host species that might make some individuals exceptional transmitters (i.e. individual super-spreaders or super-dispersers) and instead averaged over these effects by considering the total contribution of host species. Second, if social/behavioural structure prevents infectious pools from being shared homogenously among species, untargeted control of key hosts might be more effective than we predict. Finally, although we focused on gastrointestinal parasites, our framework could be extended to vector-borne infections where the number of infected vectors arising from each host species can be measured. For directly transmitted microparasites such as *Escherichia coli* and avian influenza, where shedding of infective stages can also be quantified; extensions might require additional assumptions linking shedding of infectious particles and transmission (Matthews *et al.* 2006; Brown *et al.* 2008).

Because our empirical study used natural variation among host species rather than a longer term experimental manipulation of host communities, we faced several limitations. First, unequal detection and sampling of host species might bias detection of rare supershedders towards the most studied species, leading to mis-identification of key hosts. We suspect that our calculations were not significantly affected by such differences because key hosts were distributed relatively evenly among host species that varied in densities. For example, mice were the most abundant species in all of our sites, but were key hosts for only 2/8 parasites that also infected non-mouse species (Fig.3). Hence, we do not simply conclude that the most abundant hosts are the key hosts, as might be expected if oversampling greatly biased our results. Moreover, even when we consider the possibility of dramatic variation in capture probabilities using the metrics developed in Box 1, we are still often able to differentiate the relative efficacy of different control strategies (Fig. S6). Second, although parasite communities are likely to be temporally dynamic, the broad geographic scale of our sampling required a snapshot approach. We minimised this potential bias by sampling all sites within the 2.5-month period when parasite prevalence was known to be highest at the Virginia trapping sites (Pedersen 2005). While this case study was adequate to demonstrate how our framework may guide control strategies, further applications would clearly require greater spatial and temporal resolution in host distribution, capture probabilities and parasite infection patterns. Moreover, such applications would need to consider the logistical feasibility of achieving species-specific or infection-specific control.

In conclusion, our results show that heterogeneities in transmission potential among host species often result in the formation of key host species that contribute disproportionately to parasite transmission and differentiating the distinct processes that contribute to this heterogeneity may direct the management of multihost parasites. Controlling multihost parasites is a pressing topic in the ecology of infectious diseases due to the ubiquity of such systems in nature and the implications of disease emergence by cross-species transmission for human and animal health (Cleaveland *et al.* 2001; Smith *et al.* 2009). We highlight the need to identify key hosts and tailor management efforts to the specific mechanisms driving their importance to alleviate disease risks in humans, domestic animals and wildlife through cross-species transmission and provide a foundation for future studies to do so.
